# Etidronate prevents dystrophic cardiac calcification by inhibiting macrophage aggregation

**DOI:** 10.1038/s41598-018-24228-y

**Published:** 2018-04-11

**Authors:** Carolin Bauer, Olivier le Saux, Viola Pomozi, Redouane Aherrahrou, Rene Kriesen, Stephanie Stölting, Annett Liebers, Thorsten Kessler, Heribert Schunkert, Jeanette Erdmann, Zouhair Aherrahrou

**Affiliations:** 1 Institute for Cardiogenetics, Universität zu Lübeck; DZHK (German Centre for Cardiovascular Research), Partner Site Hamburg/Kiel/Lübeck, Germany, University Heart Centre Lübeck, 23562 Lübeck, Germany; 2 Department of Cell and Molecular Biology, John A. Burns School of Medicine, University of Hawaii, Honolulu, HI USA; 3 Center for Public Health Genomics, Department of Biomedical Engineering, University of Virginia, Charlottesville, VA USA; 4 Deutsches Herzzentrum München, Technische Universität München and DZHK (German Centre for Cardiovascular Research); Partner Site Munich Heart Alliance, Technische Universität München, München, Germany

## Abstract

Cardiovascular calcification is associated with high risk of vascular disease. This involves macrophage infiltration of injured vascular tissue and osteoclast-related processes. Splenic monocytes from mice, that are predisposed (C3H) or resistant (B6) to calcification, were isolated and differentiated *in vitro* with M-CSF to generate macrophages, which aggregate to form multinucleated (MN) cells in the presence of RANKL. MN cell formation was significantly decreased in monocytes from resistant compared with calcifying mice. Conditioned media from C3H macrophages strongly induced calcification *in vitro*. However, medium from B6 macrophages inhibited calcification. An increase in ICAM-1 was detected in conditioned media from C3H macrophages compared with B6, suggesting a key role for this molecule in calcification processes. Due to natural genetic loss of *Abcc6*, the causal gene for cardiac calcification, C3H mice have reduced plasma levels of inorganic pyrophosphate (PPi), a potential calcification inhibitor. Supplementation of C3H mice with PPi or Etidronate prevented but did not completely reverse cardiac calcification. Our data provide strong evidence of the pathogenesis of macrophages and MNs during tissue calcification and suggest PPi or its analogue Etidronate as a potential inhibitor of MN formation and calcification. Furthermore, the adhesion molecule ICAM-1 was shown to play a key role in calcification.

## Introduction

Pathological calcification is defined as ectopic calcification in which calcium phosphate deposits form in soft tissues^1,2^. Soft tissue calcification occurs in many pathological processes, including atherosclerosis, diabetes, and chronic kidney disease^3,4,5^. When such calcification is associated with injured, damaged, or necrotic tissue, it is referred to as dystrophic calcification; calcification of necrotic myocardium is referred to as dystrophic cardiac calcification (DCC)^6^.

*ABCC6* (also called *MRP6*), which encodes a membrane transporter protein that inhibits ectopic calcification, is mainly expressed in hepatocytes and, to a lesser extent, in the kidney^7^. Variants of *ABCC6* were recently identified as causative for pseudoxanthoma elasticum (PXE)^8^ and some cases of generalized arterial calcification of infancy (GACI) in humans^9^, as well as DCC in mice^10,11^. For many years, PXE has been considered a metabolic disorder of systemic origin affecting multiple tissues, including the heart, muscle, blood vessels, and skin, and was thought to be caused by loss of *ABCC6* function in the liver^8^. Recently, Jansen and co-workers confirmed this hypothesis; in addition, they showed that ABCC6 facilitates the cellular efflux of ATP, which is rapidly converted into inorganic pyrophosphate (PPi), a potent inhibitor of calcification^7,12^.

Several inbred strains of mice, including DBA/2, BALB/c, 129S1/SvJ, and C3H/He, are naturally deficient in *Abcc6* due to a single nucleotide polymorphism. These mice are susceptible to DCC^13,14,15^ as well as PXE-like ectopic calcification^16^. By contrast, C57BL/6 (B6) mice harbor a wild-type *Abcc6* gene and are resistant to calcification. Previously, we used C3H/He (C3H) mice and the congenic B6.C3H^Dyscalc1^ (Cg1) mice as models to study the pathological processes leading to calcification in soft tissues, particularly the myocardium^13,14^.

We recently reported that macrophages infiltrate the necrotic tissue in the heart after cardiac injury. This macrophage infiltration into the necrotic tissue was accompanied by increased expression of markers of osteogenesis, such as *CTSK* (cathepsin K), *TRAP* (tartrate-resistant acid phosphatase), *RUNX2* (Runt-related transcription factor 2), *NF-κB* (nuclear factor kappa B), and *OPN* (osteopontin), at sites of inflammation^14^, suggesting a causal role for macrophage-derived multinucleated cells (MN) and osteoclast-like cells (OCLs) in soft tissue calcification processes.

The MN cells are formed by the fusion of mononuclear progenitors of the monocyte (Mo)/macrophage (Ma) lineage^17^. MN cells can exhibit different phenotypes depending on the surrounding micro-environment^18^. Giant cells are associated with granulomatous diseases and tumors, whereas OCLs play important roles in defense and tissue remodeling. Although distinct, the aforementioned types of MN cells share the same functional markers, and both differentiate by fusion of precursor cells of the Mo/Ma lineage. Indeed, fusion is an obligatory step in the structural and functional differentiation of these cells. Two key molecules are essential for promotion of osteoclastogenesis, including macrophage colony-stimulating factor (M-CSF) and receptor for activation of NF-κB (RANK) ligand (RANKL)^19,20^. The RANKL/RANK/Osteoprotegerin (OPG) system, an important pathway in vascular calcification, links the vascular, skeletal, and immune responses. RANKL, which is highly expressed by T cells and osteoblasts (OBs), binds to RANK, a transmembrane receptor on the surface of monocytes and macrophages. In bone tissue, OB expression of RANKL together with M-CSF is essential for the complete development of MN bone-resorptive osteoclasts (OCs) from monocytic precursors. However, OPG blocks the RANKL-mediated differentiation of OCs from OBs. Mice lacking Opg exhibit severe osteoporosis and arterial calcification, suggesting that osteoclastogenesis shares many features with the processes of vascular and skeletal calcification^19^. However, the exact roles of these macrophage-derived MN cells in DCC remains unclear.

Intercellular adhesion molecules stimulate monocyte adhesion and migration. Thus, ICAM-1- activity (intercellular adhesion molecule 1) triggers atherosclerotic plaque development by enhancing the inflammatory response^21^. High levels of soluble ICAM-1 in the plasma have been associated with cardiovascular disease, suggesting a possible role for ICAM-1 as a biomarker of vascular injury^22^.

In addition, the complement system is linked to osteogenesis as a trigger for inflammatory responses, and it also influences OB–OC interactions. The complement system contains two major components, C3a and C5a, that promote MN cell differentiation in the absence of RANKL/M-CSF^23^. Activation of C3a and C5a leads to macrophage and T cell activation via their corresponding receptors, C3ar and C5ar. C3a induces OB differentiation^24^, whereas C5a is involved in OB migration^23^. Both C5ar and C3a are upregulated during osteogenic differentiation, whereas C5a expression is not detectable in OCs derived from peripheral blood mononuclear cells.

Abcc6 deficiency leads to a reduction of PPi levels in plasma in mice and PXE patients^12^. We demonstrated very recently that supplementation of *Abcc6* KO mice with PPi or bisphosphonate etidronate inhibits cardiac calcification and PXE-like spontaneous calcification^25^. Bisphosphonates, such as etidronate, are known potential inhibitors of osteoclastogenesis^26^. Also bisphosphonates, stable compounds derived from pyrophosphate, are used in humans for the treatment of osteoporosis and bone metastasis^27^. The downstream effects of PPi deficiency are unclear. Here, we studied MN cell formation and the expression of key molecules associated with macrophage aggregation, adhesion, inflammation, and the complement system *in vivo* and *in vitro*. Furthermore, we tested the effect of administration of PPi, a potent calcification inhibitor, on the formation of MN cells *in vitro* and the pathogenesis of *DCC* in calcifying C3H/He mouse model.

## Material and Methods

### Animals

Female mice from the C57BL/6 J (B6) and C3H/HeJ (C3H) inbred strains were purchased from Charles River Laboratories and maintained for at least 2 weeks prior to use in experiments to allow them to acclimate to the environmental conditions of the vivarium. Congenic B6.C3H^Dyscalc1^ were generated as reported previously^13,14^. *Abcc6*^−/−^ mice were generated on the 129/Ola background and backcrossed onto a C57BL/6 J background at least 10 times^28^.

### Freeze–thaw injury

Animal studies were performed in accordance with institutional guidelines and regulations. The animal studies committee of Schleswig-Holstein, Germany and the University of Hawaii Institutional Animal Care and Use Committees approved these studies.

Animals were housed and fed according to good animal attendance practice. Freeze–thaw injury was performed as reported previously^13,14^. After 7 days, mice were sacrificed under anesthesia by cervical dislocation. Hearts were excised, and calcium phosphate deposits were evaluated using the Randox calcium kit (Randox Laboratories, Crumlin, UK), as previously described^28^.

### Cell culture and *in vitro* osteoclastogenesis

Three mice in each group were sacrificed at the age of 8–10 weeks under anesthesia by cervical dislocation, and spleens were collected. Monocytes were isolated from the spleens, suspended in cell culture medium (DMEM) containing 10% FBS, and placed in 8-well chamber slides (1.5 × 10^6^ cells/cm^2^). After 24 hours, 75 ng/μl recombinant M-CSF (Miltenyi Biotec GmbH, Bergisch Gladbach, Germany), a factor required for macrophage differentiation, was added. On days 6 and 9, the culture medium was removed and replaced with fresh medium containing M-CSF and RANKL (75 ng/μl; BioLegend, San Diego, CA, USA) to obtain macrophage-derived MN cells. Successful differentiation was confirmed after 12 days of culture by staining with a TRAP staining kit (TaKaRa Bio, USA). Nine distinct pictures of each well (1 cm^2^) were taken through a microscope. The number of TRAP-positive cells with more than three nuclei in each well was counted and expressed as number of MN cells/Well.

### Treatment of macrophages with platelet-free mouse plasma or PPi

To obtain platelet-free plasma, whole mouse blood was collected from mice in 1.5 ml CTAD anti-coagulation tubes and depleted of platelets by filtration through a Centrisart 300,000 kDa cut-off filter (Sartorius, Göttingen, Germany). Samples were stored at −20 °C until use.

Monocyte-derived macrophages were stimulated for 6 days with cell culture medium containing M-CSF/RANKL. Macrophages were then treated with the indicated concentrations of PPi (Sigma-Aldrich, St. Louis, MO, USA) or platelet-free plasma from DCC-susceptible or -resistant mice. The medium was replaced on day 9, with the addition of fresh plasma. On day 12, cells were fixed and stained using the TRAP kit to detect MN cells. TRAP-positive cells with more than three nuclei were counted under a microscope.

### Collection of conditioned media from MN cells

Monocytes were isolated from the spleens of C3H and B6 mice and differentiated into macrophage-derived MN cells, as described above. After that, cells were washed three times in 1 × PBS and placed in fresh medium without M-CSF or RANKL. The supernatant was collected after 24 hours and used for PPi measurement or in calcification assays with C3H/10T1/2 mesenchymal stem cells, as described below.

### Induction of calcification in C3H10T1/2 cells using inorganic phosphate (Pi)

C3H/10T1/2 cells were seeded in 48-well plates (0.25 × 10^5^ cells/well), and calcification was induced using 2.6 mM inorganic phosphate (Pi). Medium was changed every 3 days until visible calcification occurred. Calcification was determined by calcein staining and quantified using the Randox calcium kit.

### qPCR analysis

Total RNA was isolated from myocardial tissue or cultured cells using the RNeasy kit (Qiagen, Valencia, CA, USA). After reverse transcription into cDNA, mRNA levels were determined by relative quantitative RT-PCR and analyzed by the ΔΔC_T_ method, as described previously^11,14^. The primers used for gene expression studies are listed in Table 1. β-*actin* was used as an internal standard.

**Table 1:** List of primer pairs used for qPCR analysis.

Gene Symbol	Forward primer	Reverse primer
*Ctsk*	AGGGAAGCAAGCACTGGATA	AAGCTGGCCATGTTGGTAAT
*Trap*	ACTTGCGACCATTGTTAGCC	CTTTGCTTGATGTCGCACAG
*Runx2*	AAGTGCGGTGCAAACTTTCT	ACGCCATAGTCCCTCCTTTT
*C3a*	GCATCTTGCCTTTGTCTTGG	CTCAAACTCTGCGGAGAAGA
*C3ar*	AGTCCTGGAGCCTTTGGATT	TCTTGGGGTTGAAACAGAGG
*C5a*	TGGCTACACTGAAGCATTTGA	CGTGAGAGACTGGGCTTTCT
*C5ar*	GGTTACCACAGAACCCAGGA	CCATCCGCAGGTATGTTAGG
*NOS2* (*iNOS*)	CAGAGGACCCAGAGACAAGC	CCTGGCCAGATGTTCCTCTA
*Arg-1*	GGAAAGCCAATGAAGAGCTG	CTGGTTGTCAGGGGAGTGTT
*CD68*	AGGACCGCTTATAGCCCAAG	ATTTCCGTGACTGGTGGTG
*ICAM-1*	GAGAGTGGACCCAACTGGAA	TGAGGTCCTTGCCTACTTGC
*ß-Actin*	ACTGGGACGACATGGAGAAG	GGGGTGTTGAAGGTCTCAAA

### ICAM-1 ELISA

ICAM-1 levels in blood plasma and conditioned macrophage media were quantified according to the manufacturer’s instructions using the mouse ICAM-1/CD54 Quantikine ELISA kit (R&D Systems, Minneapolis, MN, USA).

### PPi measurement

Blood samples for PPi measurement were collected via cardiac puncture (using 27 G needles). Blood was kept on ice and centrifuged for 10 min at 1000 g at 4 °C. Plasma was transferred into separation tubes (Sartorius Centristart 1, 300,000 MW, 13279E) and filter-centrifuged at 2000 g for 25 min at 4 °C. Filtered plasma samples were stored at −80 °C until analysis.

For PPi measurement monocytes were seeded and differentiated in 24-Well plates (1.5 × 106 cells/cm^2^). At day 7, 300 µl FBS-free cell culture medium was added to each well. The conditioning media was collected after 24 hours.

To measure PPi concentration, PPi was first converted to ATP by Adenosine 5′ Triphosphate Sulfurylase (ATPS) (ProSpec, ENZ-353) in the presence of excess Adenosine 5′-phosphosulfate (APS) (Santa Cruz, sc-214506). The concentration of ATP generated from PPi was measured using the BacTiter-Glo kit (Promega, G8230).

Basal ATP level was also measured in plasma samples and was found negligible.

### Treatment of *Abcc6*^−/−^ and C3H mice with PPi or etidronate

The animal studies committee of Schleswig-Holstein, Germany and the University of Hawaii Institutional Animal Care and Use Committees approved these studies.

A stock solution of PPi (5 mg/ml) was prepared in saline (0.9% NaCl) or PBS. For C3H mice, a dose of 20 mg/kg PPi was administered via intraperitoneal (i.p.) injection 1 day before the induction of calcification by freeze–thaw injury. Immediately after freeze–thaw injury, a 2X dose of PPi was administered. Thereafter, mice were injected i.p. every day with 20 mg/kg PPi. For *Abcc6*^−/−^ mice, the same dosing schedule was followed, but PPi was administered at 12 or 25 mg/kg. Etidronate was purchased (TCI Deutschland GmbH, Germany) and administered daily at a dose of 2 mg/kg, as reported previously^25^. Control animals received injections of saline or PBS.

### Statistical analysis

Data are presented as the mean ± SEM. Unpaired t-tests were performed using GraphPad. P < 0.05 was considered statistically significant. For all tests: *P < 0.05; **P < 0.01; ***P < 0.001; ****P < 0.0001; and n.s., not significant.

## Results

### Elevated MN cell formation in mice predisposed to calcification

**Figure 1:**
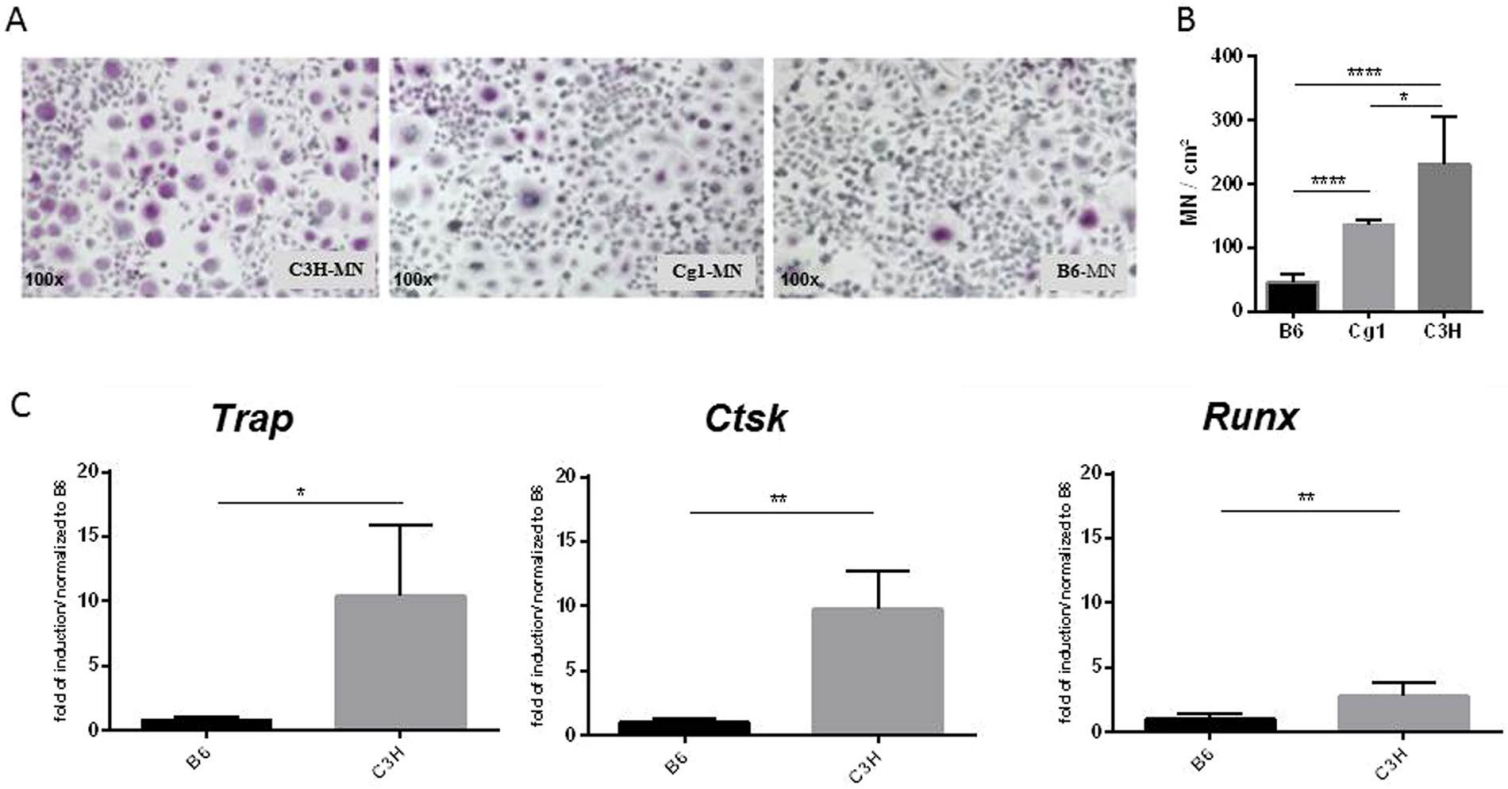
Elevated multinucleated (MN) cell formation in mice predisposed to calcification. (**A**,**B**) Monocytes from DCC-resistant B6, DCC-susceptible C3H and Cg1 mice were isolated and subjected to MN differentiation with M-CSF and RANKL. (**B**) TRAP-positive cells with more than three nuclei were counted and quantitated. (**C**) Relative quantitation of the osteogenic markers *Trap*, *Ctsk*, and *Runx2* in MN cells from both B6 and C3H mice.

Monocytes from the spleens of C3H/He and C57BL/6 mice were isolated and differentiated *in vitro*, first into macrophages using M-CSF, and then into MN cells by treatment with RANKL. MN cell formation was compared between macrophages from calcification-susceptible and -resistant mice. More MN cells formed from macrophages from C3H mice than from macrophages from B6 mice (Fig. 1A,B).

*Abcc6* (which encodes the Mrp6 protein) was previously identified as a gene responsible for calcification in predisposed mice^10^. Specifically, a polymorphism in *Abcc6* reduces the levels of Mrp6, rendering the mice prone to calcification^11^. To determine whether the observed differences in MN formation were due to variations in *Abcc6* deficiency, MN formation was analyzed in macrophages from B6.C3H^Dyscalc1^ (Cg1) mice, congenic mice containing the *Abcc6* locus from DCC-susceptible C3H mice on a DCC-resistant B6 genetic background^13,14^. More MN cells formed from Cg1 than from C57BL/6 macrophages (Fig. 1A,B).

To confirm this finding at the molecular level, levels of the osteogenic markers *Trap*, *Ctsk* and *Runx2* were measured. All three markers were more highly expressed in macrophage-derived MN cells from mice predisposed to calcification than in those from the DCC-resistant B6 mice (Fig. 1C).

### Identification of MN cells at sites of calcification after injury in calcification-prone mice

**Figure 2:**
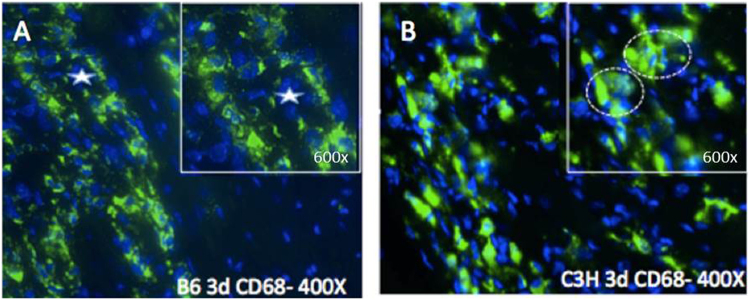
Aggregation and fusion of macrophages in heart tissue of C3H mice at the early stage (day 3) after injury. Infiltrating macrophages in the heart tissue of B6 (**A**) and C3H mice (**B**) stained for the macrophage marker CD68 by immunohistochemistry. Nuclei were stained blue with DAPI. Circles indicate multinucleated aggregates in C3H mice.

DCC-susceptible C3H and DCC-resistant B6 mice were used as models for calcification *in vivo*. Our group previously developed a freeze–thaw injury model of acute calcification in mice. On day 1, monocytes/macrophages could be detected infiltrating the injury site in the area between necrotic and healthy tissue. On day 3, macrophages had infiltrated most of the necrotic tissue, as shown by immunofluorescence staining for CD68 (Fig. 2A,B). As early as day 3, calcium phosphate deposits could be visualized by calcein staining, as reported previously^14^. Close inspection of the cell morphology revealed macrophage aggregations within the calcified regions of the cardiac tissue in C3H mice (Fig. 2B, circles), but not in the non-calcified cardiac tissue in B6 mice (Fig. 2A). Thus, macrophages from C3H mice are prone to aggregation and show osteogenic features, as reported previously by our group^14^.

### Calcification-prone mice are prone to macrophage polarization

**Figure 3:**
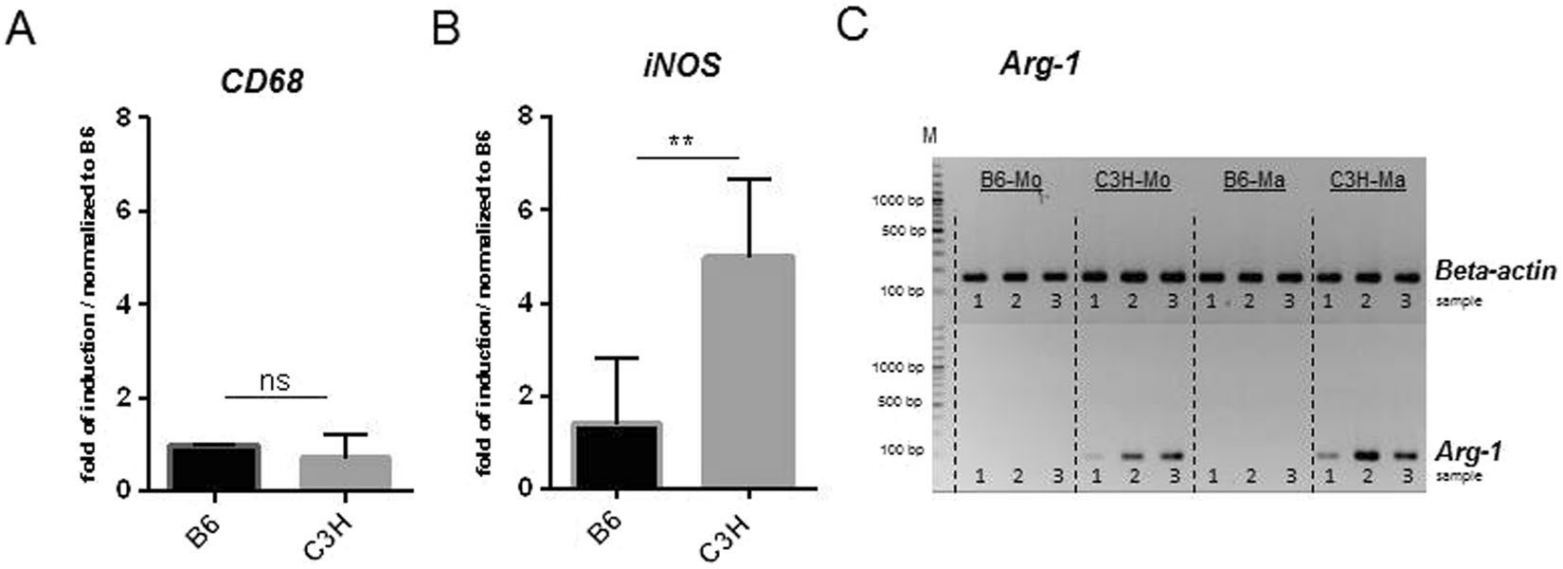
Gene expression analysis in monocytes and macrophages. (**A**,**B**) Relative qPCR analysis of *CD68* and *iNO*S. (**C**) RT-PCR analysis of *Arg-1* and *ß-actin* in monocytes and macrophages of B6 and C3H mice. Images of *Arg-1* and *ß-actin* were taken from 2 different parts of the same gel. The Numbers 1 to 3 stand for sample number. Expression of *CD68* was used as a marker for the macrophage lineage, while *iNOS* expression was used to indicate M1 polarization, and *Arg-1* expression was used to indicate M2 polarization (**A**,**C**). Monocyte-derived macrophages from B6 and C3H mice expressed equal levels of *CD68*, indicating equivalent differentiation to the macrophage lineage (**A**). C3H macrophages expressed higher levels of the M1 marker *iNOS* (**B**) and the M2 marker *Arg-1* than B6 macrophages. No expression of *Arg-1* was detected in B6 macrophages (**C**).

To characterize the phenotype of the macrophages that mediate DCC, the expression of CD68, a typical marker for the macrophage lineage, was evaluated in spleen-derived macrophages along with the expression of inducible nitric oxide synthase (iNOS) and arginase-1 (*Arg-1*), markers of M1 and M2 macrophages, respectively (Fig. 3). No differences in the expression of *CD68* were observed between monocyte-derived macrophages from B6 and C3H mice (Fig. 3A). However, C3H macrophages were more prone to polarization and expressed higher levels of M1 and M2 markers than B6 macrophages (Fig. 3B,C). Expression of *Arg-1* was detected only in macrophages derived from splenic monocytes from C3H mice (Fig. 3C).

### Higher *C3a* and *C3ar* expression in MN precursor cells from DCC-susceptible mice

**Figure 4:**
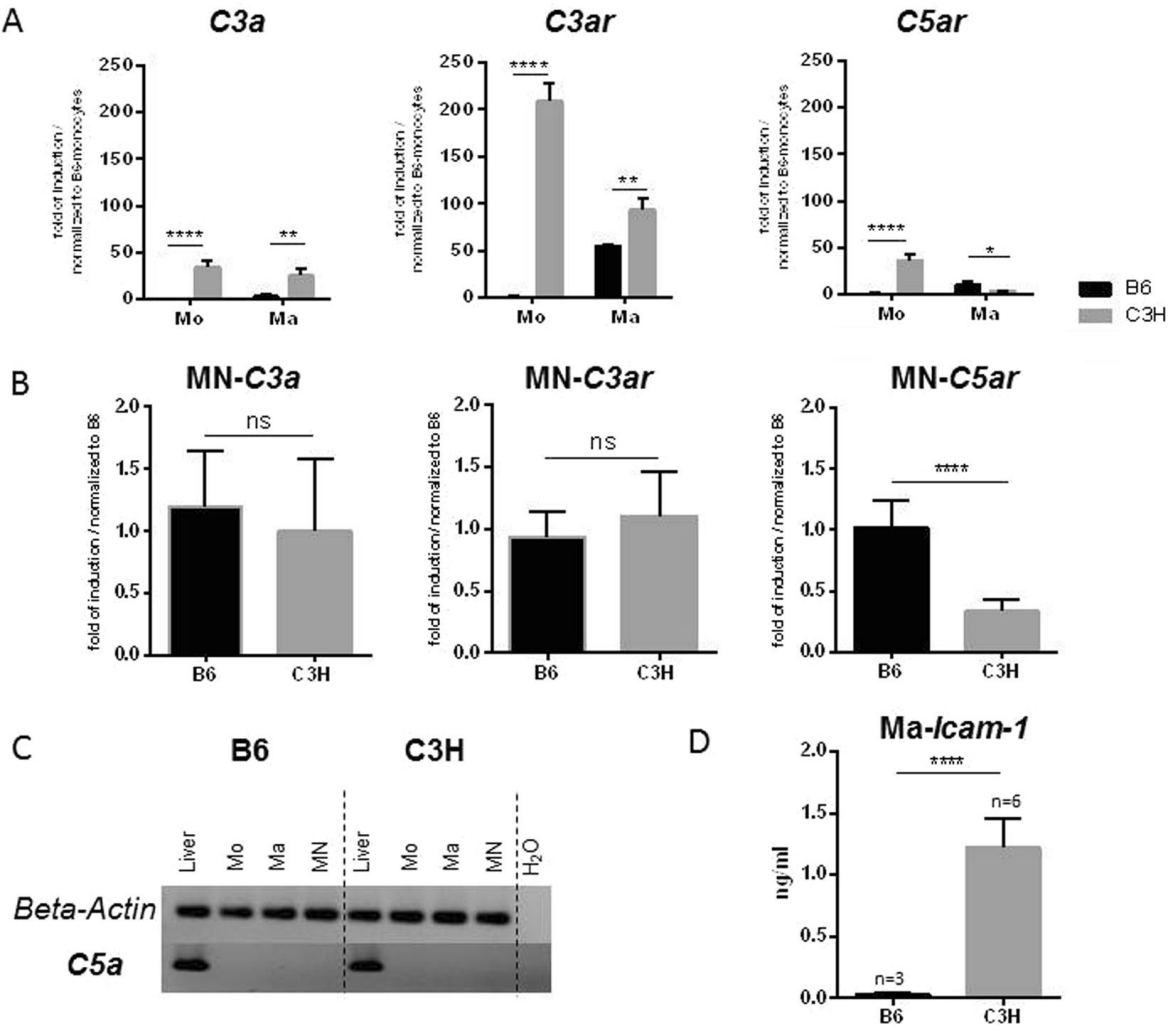
Expression of complement components and receptors and ICAM-1. (**A**) *C3a*, *C3ar*, and *C5ar* expression in monocytes and macrophages. (**B**) Gene expression analysis in MN cells. (**C**) Images of *C5a* and *ß-actin* were taken from 2 different parts of the same gel. *C5a* expression was not detected by RT-PCR in monocytes, macrophages, or MN cells of B6 or C3H mice. Liver tissue was used as a positive control. In DCC-susceptible C3H mice, *C3a* and *C3ar* were highly expressed in the early stages of cell differentiation, but expression of *C3* increased much more slowly during macrophage differentiation. *C3a* and *C3ar* expression in MN cells was not significantly different between the strains. No expression of *C5a* was detected in either group (**C**), although the MN cells of B6 mice tended to express higher levels of *C5ar* (**B**,**D**) ICAM-1 release from macrophages of B6 and C3H mice was analyzed by ELISA. Higher release of ICAM-1 was observed from the macrophages of calcification-susceptible C3H mice. (**E**) Expression of ICAM-1 in calcifying myocardial tissue from C3H mice compared with non-calcifying tissue from B6 mice following freeze–thaw injury.

Complement factors *C3a* and *C5a* play key roles in osteogenesis^23^. Accordingly, the expression of both factors and their corresponding receptors was evaluated during the differentiation of MN from monocytes of both B6 and C3H mice (Fig. 4). Monocytes and macrophages from C3H mice expressed higher levels of *C3a* and C3ar than those from B6 mice (Fig. 4A), but *C3a* and *C3ar* expression in MN cells did not differ between the strains (Fig. 4B). No expression of *C5a* could be detected in either group (Fig. 4C), although the MN cells of B6 mice tended to express higher levels of *C5ar* (Fig. 4B).

### Higher release of ICAM-1 by macrophages of C3H mice

Splenic monocytes from B6 and C3H mice were differentiated into macrophages with M-CSF. The medium was then changed, and culture supernatants were collected 2 days later. The intercellular adhesion molecule ICAM-1 plays a key role in cell–cell adhesion and aggregation as well as in inflammation and osteoclastogenesis. The level of ICAM-1 in culture supernatants from B6 and C3H macrophages was compared (Fig. 4D). A high level of ICAM-1 was detected in culture supernatants from C3H monocyte-derived macrophages, while very little ICAM-1 was released by B6 macrophages.

### Blood serum factors from DCC-resistant mice inhibit MN cell differentiation

**Figure 5:**
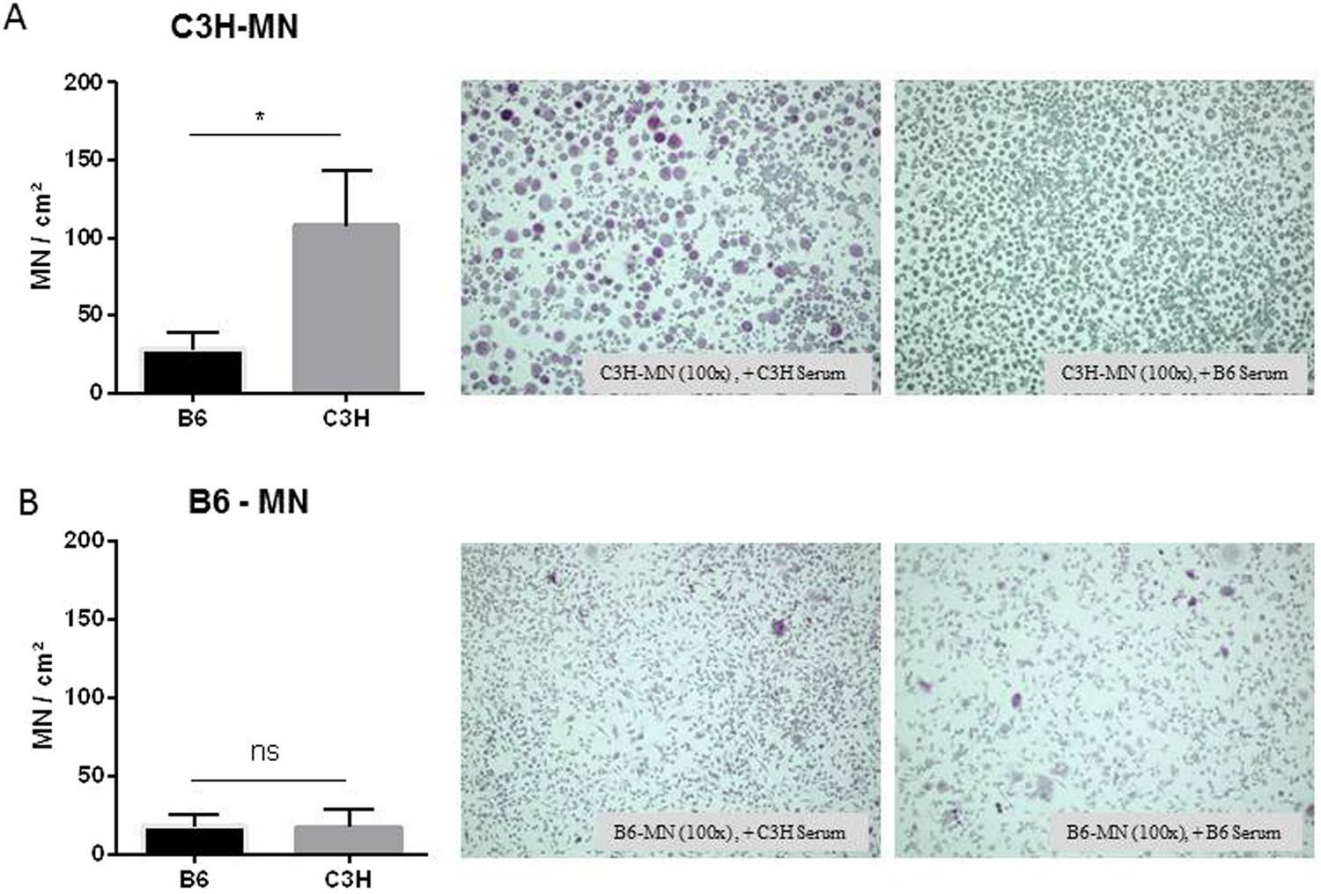
Serum factors from DCC-resistant B6 mice inhibit MN cell formation in DCC-susceptible C3H mice. Splenic monocytes from C3H (**A**) or B6 (**B**) mice were differentiated into MN cells, and the serum in the culture supernatant was replaced with plasma from either C3H or B6 mice. MN formation was analyzed microscopically by TRAP staining. TRAP-positive cells with more than three nuclei were counted and quantified.

DCC is considered a systemic disorder, and circulating factors can regulate the initiation and development of the disease. Accordingly, we investigated whether circulating factors could influence MN cell formation *in vitro*. Splenic monocyte-derived macrophages were treated with serum from calcification-prone C3H and calcification-resistant B6 mice. The serum of B6 mice inhibited the formation of MN cells from macrophages of C3H mice (Fig. 5A,B). However, serum from DCC-susceptible C3H mice had no effect on the formation of MN cells from macrophages of B6 mice (Fig. 5B).

### Reduced PPi levels and increased ICAM-1 expression in C3H mice

**Figure 6:**
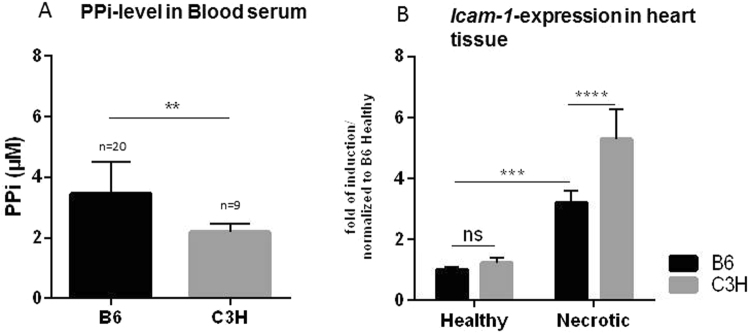
(**A**) Plasma levels of inorganic pyrophosphate (PPi) and *ICAM-1* expression in injured myocardial tissue. (**A**) DCC-resistant B6 mice have higher plasma PPi levels than DCC-susceptible C3H-mice. (**B**) Gene expression analysis demonstrates higher *ICAM-1* expression in calcifying myocardial tissue from C3H mice than in non-calcifying tissue from B6 mice following freeze–thaw injury.

Jansen *et al*. showed recently that ABCC6 facilitates the cellular efflux of ATP, which is rapidly converted into PPi, a potent inhibitor of calcification^12^. In addition, they also reported reduced plasma PPi levels in *Abcc6*-deficient mice as well as in human PXE patients. Here, we tested the levels of plasma PPi in C3H and B6 mice. C3H mice, which are prone to calcification, had reduced plasma PPi compared with B6 mice (Fig. 6A).

Due to the high level of ICAM-1 released *in vitro* by macrophages from calcification-prone C3H mice, the differential expression of this gene was evaluated *in vivo*, in necrotic myocardial tissue from DCC-susceptible C3H and DCC-resistant B6 mice. Although necrotic tissue and macrophage infiltration were observed in both strains of mice, a marked increase in *ICAM1* expression was observed at day 3 after injury in the calcification-susceptible C3H mice (Fig. 6B).

### PPi inhibits MN cell formation

**Figure 7:**
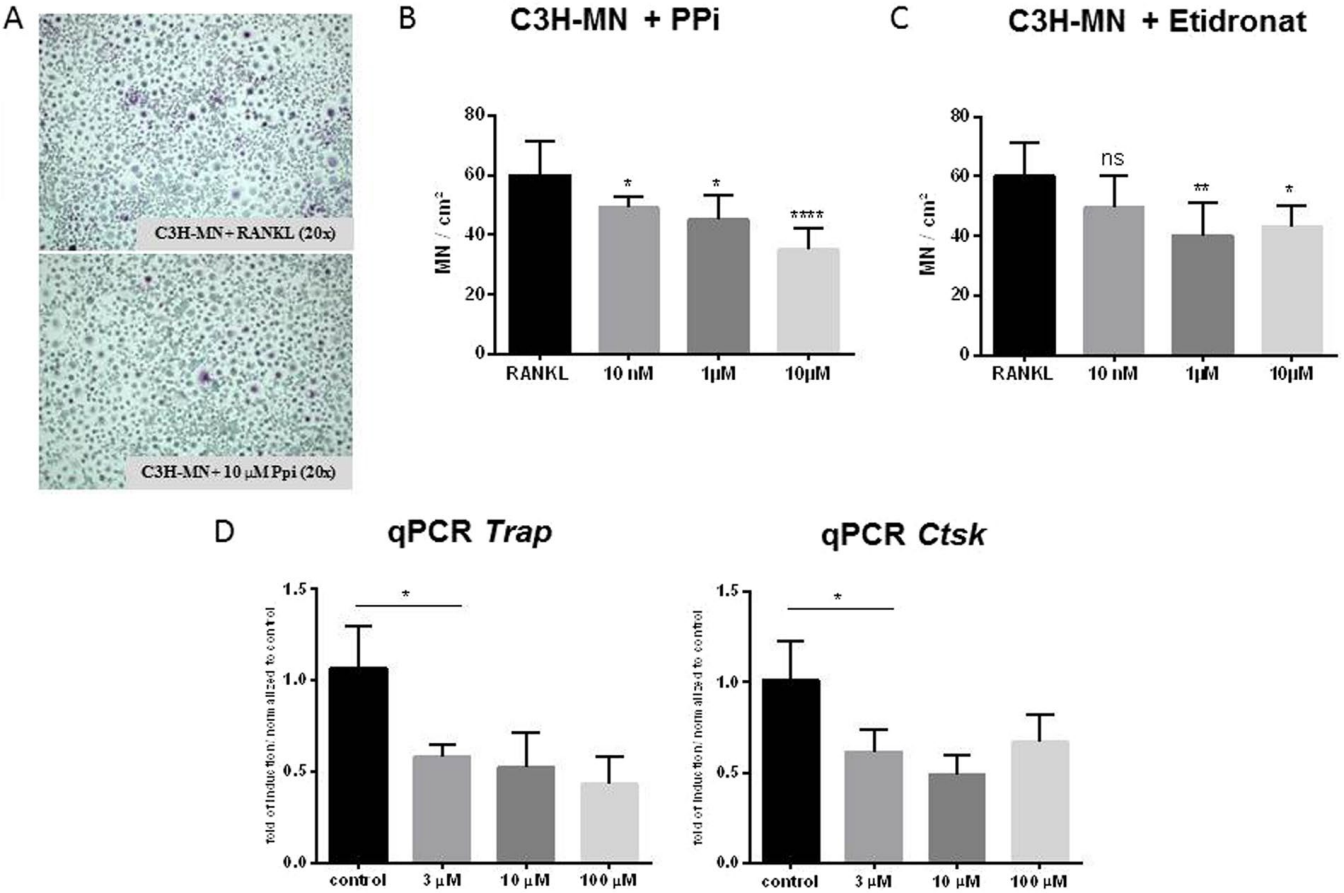
Effect of PPi and etidronate on MN cell formation. (**A**,**B**) Monocytes from DCC-susceptible C3H mice were isolated and subjected to MN differentiation with M-CSF and RANKL. At day 6, the indicated concentrations of PPi were added. MN cell formation was imaged (**A**) and quantitated (n = 8 in each group; (**B**). Relative quantitation of the osteogenic markers *Trap* and *Ctsk* in MN cells differentiated in the presence of the indicated concentrations of PPi (n = 3 in each group; (**C**).

Very recently, Abcc6 was linked to extracellular production^29^ of PPi, which inhibits calcification in smooth muscle cells *in vitro*^30^. Consistent with this, DCC-resistant wild-type C57BL/6 mice had higher plasma PPi levels than DCC-susceptible *Abcc6*-deficient mice^7^. We therefore investigated whether PPi could inhibit MN cell formation. A range of PPi concentrations, from 3 to 100 μM, were tested for potential inhibition of MN cell formation from C3H macrophages. PPi significantly decreased MN cell formation in a dose-dependent manner (Fig. 7A,B). Moreover, *Ctsk* and *Trap* were downregulated following PPi treatment (Fig. 7C).

### Effect of conditioned media from macrophages and MN cells on calcification

**Figure 8:**
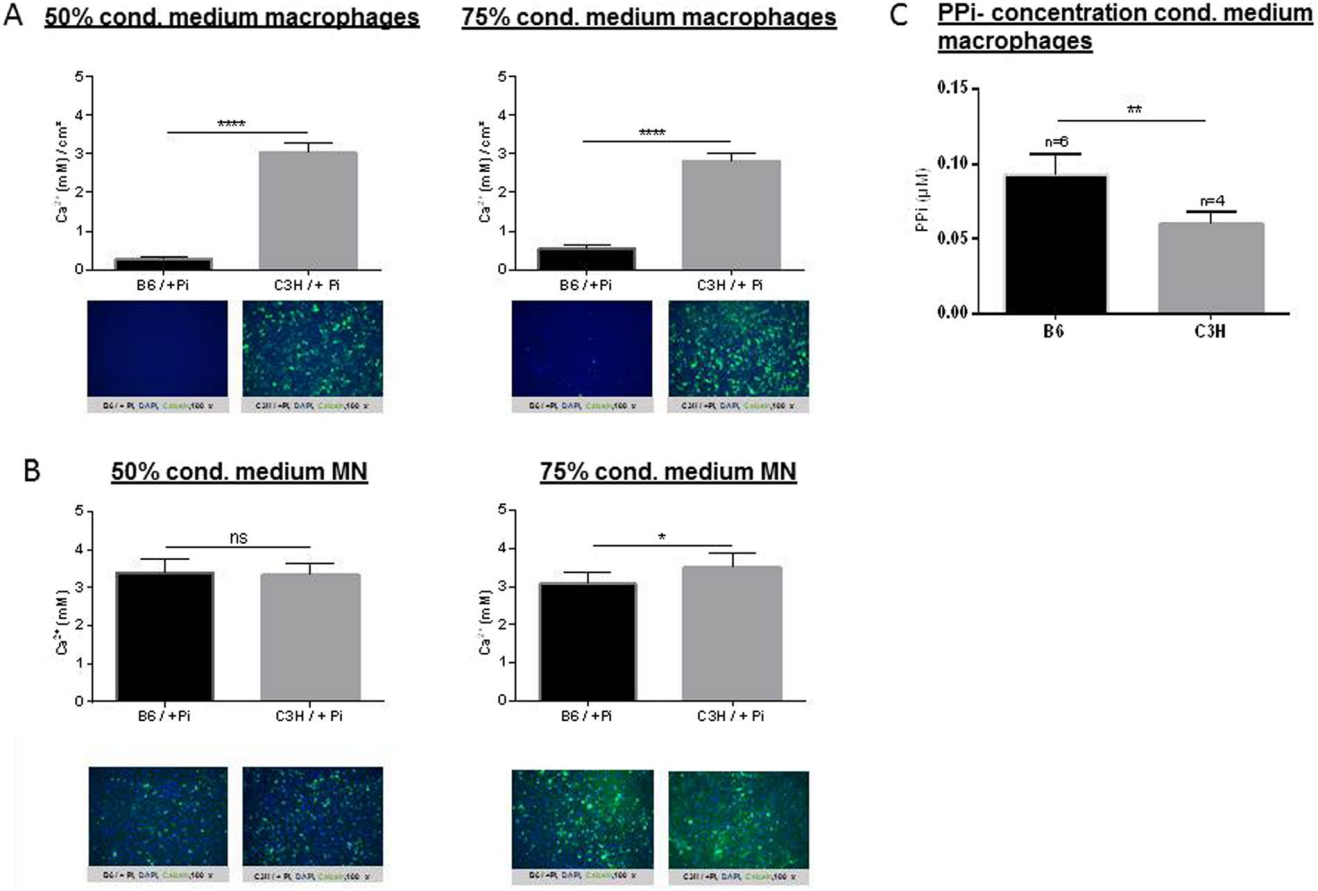
Effect of medium conditioned by macrophages (**A**) and MN cells (**B**) on calcification. Monocytes from DCC-susceptible C3H mice were isolated and subjected to MN differentiation using M-CSF and RANKL. At day 6, conditioned media from the macrophages was collected and PPi-concentration was measured (**C**). At day 12, after MN cell differentiation, cells were washed with PBS and fresh medium was added, and conditioned media from MN cells was collected 1 day later. Calcification was induced in C3H/10T1/2 mesenchymal stem cells with 2.6 mM Pi in the presence of conditioned media from macrophages or MN cells from C3H or B6 mice (diluted 50% or 75%). Calcium deposits were quantitated and compared. Nuclei are shown by DAPI staining, in blue, and calcified deposits are shown by calcein staining, in green.

To test whether macrophages or MN cells from C3H mice secreted factors that could induce osteogenesis, conditioned media from macrophages and MN cells from DCC-susceptible C3H and DCC-resistant C57BL/6 mice was collected. This conditioned media was used together with Pi, which induces calcification in C3H/10T1/2 cells. Conditioned media from monocyte-derived macrophages from B6 mice dramatically inhibited calcification of C3H/10T1/2 cells in response to Pi (Fig. 8A). Calcification was increased when C3H/10T1/2 cells were treated with 75% conditioned media from C3H MN cells compared with medium from B6 MN cells. No effect on calcification was observed when 50% conditioned media was used (Fig. 8B). Conditioned media from B6 macrophages showed a stronger inhibitory effect on calcification than conditioned media from B6 MN cells (Fig. 8A,B). In addition PPi-concentration was measured (Fig. 8C). PPi was found reduced in the conditioned media of macrophages from C3H mice.

### PPi inhibits calcification in both C3H and *Abcc6*^−/−^ mice *in vivo*

**Figure 9:**
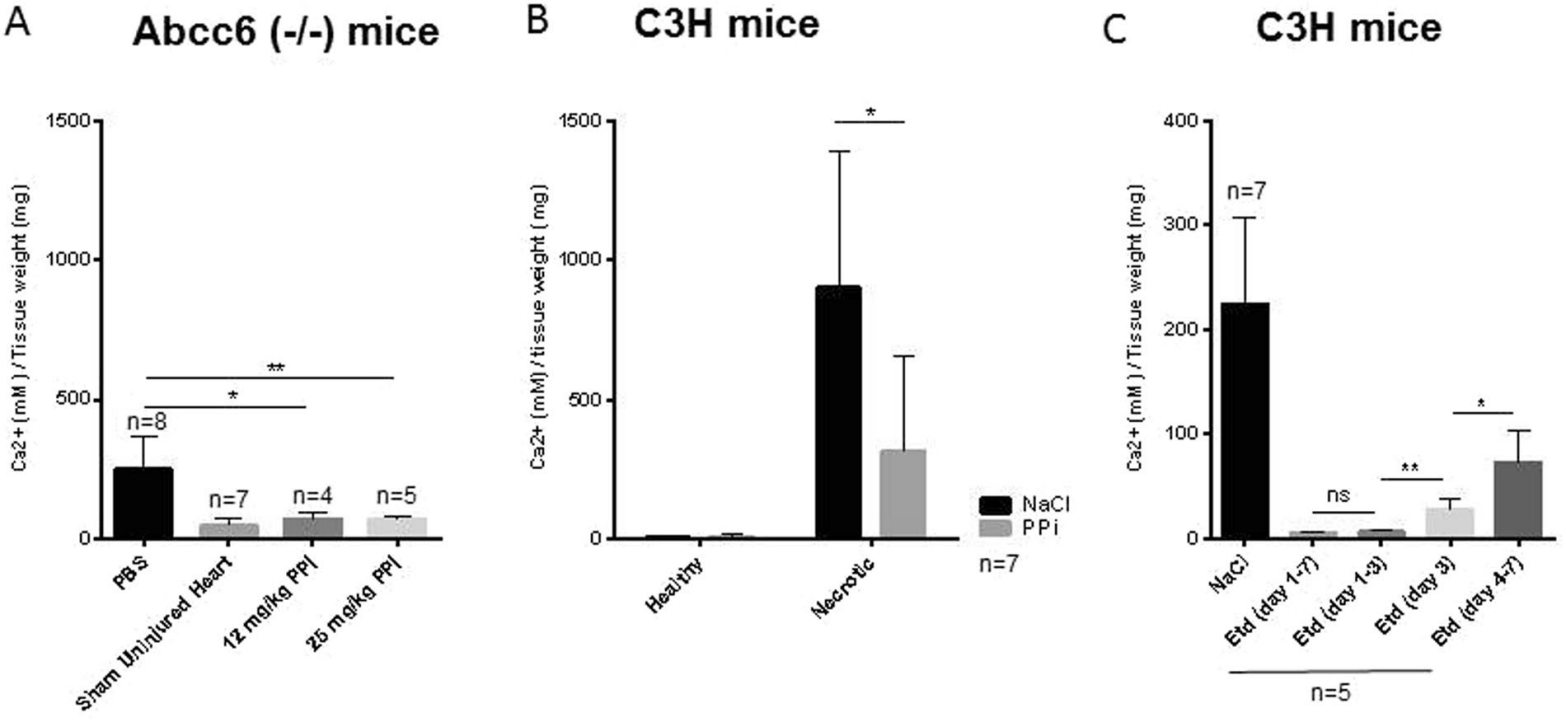
Effect of PPi and etidronate on DCC in *Abcc6*^−/−^ and C3H mice. Calcification was induced in DCC-susceptible Abcc6^−/−^ mice (**A**) and C3H mice (**B**,**C**) by freeze–thaw injury. Mice were treated with PPi (1 day before injury, then daily for 1 week) (**A**,**B**) or etidronate (Etd) (at the indicated time points) (**C**). Control mice were injected with NaCl or PBS. One week after injury, hearts were collected. Whole heart (Panel A and C) or Healthy myocardium and necrotic myocardium (Panel B) from the same heart were excised, and calcium phosphate deposits were assessed. PPi- and etidronate-treated mice were compared with PBS- or NaCl-treated (control) mice.

The *in vitro* data described above indicate that PPi is a potential inhibitor of MN cell formation, and thus may also inhibit calcification *in vivo*. To test this hypothesis, DCC-susceptible *Abcc6*^−/−^ mice and C3H mice were subjected to freeze–thaw injury to enhance calcification. For *Abcc6*^−/−^ mice, two different concentrations of PPi were used (12 and 25 mg/kg/day) (Fig. 9A). For C3H mice, a dose of 20 mg/kg/day was used (Fig. 9B). PPi was administered by i.p. injection 1 day before freeze–thaw injury, and then daily for a period of 7 days. At that time, the hearts were excised, and calcium phosphate deposition was assessed. It is known that C3H mice are more predisposed to calcification than *Abcc6*^−/−^ mice because additional loci are associated with DCC in mice^1^. As expected, calcification was elevated in the hearts of both strains of mice following freeze–thaw injury, especially in necrotic myocardium as opposed to healthy tissue (Fig. 9B). However, supplementation with PPi inhibited calcification in both *Abcc6*^−/−^ mice and C3H mice (Fig. 9A,B).

### Etidronate prevents but does not completely reverse DCC

To determine whether etidronate could prevent or reverse DCC, C3H mice in which cardiac calcification had been induced by freeze–thaw injury were treated with 8 µmol/kg etidronate. Treatment was either initiated immediately after freeze–thaw injury and continued for 3 or 7 days, or started at day 4 post-injury, after the initiation of calcification (Fig. 9C). Treatment with etidronate after freeze–thaw injury and before the initiation of calcification prevented the development of DCC, but treatment after the initiation of calcification did not fully reverse calcium deposition. A single injection of etidronate during the macrophage infiltration and aggregation phase at day 3 after injury dramatically inhibited DCC progression.

## Discussion

Soft tissue calcification shares common features with bone calcification. Loss of *Abcc6* is sufficient to cause calcification, and Abcc6 deficiency leads to a lack of PPi^7,10–12^. The cellular component of calcification is mostly due to osteogenic differentiation of infiltrating monocyte-derived macrophages; smooth muscle cells and pericytes also contribute^31–33^. Some infiltrating macrophages fuse together to form MN cells. The role of these cells in DCC is not well understood. In this study, we showed that macrophages from calcification-susceptible mice express higher levels of inflammatory markers and the adhesion molecule ICAM-1 than macrophages from calcification-resistant mice, which leads to an increase in the differentiation of MN cells towards an osteogenic phenotype, significantly contributing to calcium phosphate deposits at the site of injury. Administration of PPi or etidronate prevents but does not reverse the calcification process *in vivo*.

We showed previously that DCC is caused by mutation of *Abcc6* in C3H and other inbred strains of mice^11^. Four loci, *Dyscalc1–4*, were initially shown to be associated with DCC. Subsequently, a major locus containing *Abcc6* was fine-mapped and determined to be causal for DCC. The three other loci act as modifiers, and influence the severity of DCC^1^. Higher levels of calcium phosphate deposits were observed in C3H mice with all *Dyscalc1–4* loci than in *Abcc6*^−/−^ B6 mice or in congenic B6.C3H^Dyscalc1^ mice that only lack *Abcc6* within the *Dyscalc1* locus.

In mice and PXE patients, the effect of Abcc6 in ectopic calcification is largely due to systemic PPi deficiency^12,25^. Reduced plasma PPi levels are associated with the mineralization phenotype of several heritable or acquired conditions, including DCC^34–39^. In the present study, we confirmed that the calcification-susceptible C3H/He mouse model has reduced plasma levels of PPi. In several animal models, PPi supplementation can attenuate pathological calcification of various etiologies^40,34,37^. Because PPi has a short serum half-life, bisphosphonates (BiPs), stable non-hydrolyzable analogues of PPi, were developed for therapeutic use in osteoporosis and bone metastasis^41^. Two BiPs were tested in *Abcc6*^−/−^ mice as a model for PXE, with mixed results^42^. Alendronate had no effect, but a high dose of etidronate modestly decreased calcification in the vibrissae of *Abcc6*^−/−^ mice.

In a recent review, Orriss and co-workers described a role for PPi in osteogenic/OC differentiation as a potent inhibitor of osteoclastogenesis^43^. Indeed, the presence of osteogenic markers such as RUNX2, bone morphogenic protein (BMP)−2, and alkaline phosphatase (ALPL) in calcified tissues (skin and vasculature) is also associated with ABCC6 deficiency^44,45^.

Here, we demonstrated in two independent mouse models (C3H/He and B6.C3H^Dyscalc1^) that macrophage aggregates exhibit both OC and OB characteristics: they express the OC markers *Trap* and *Ctsk*, as well as OB markers such as *Runx2*, indicating that these cells are incompletely differentiated and appear in lesions before mature calcification. We used conditioned media from macrophages derived from both calcification-prone and calcification-resistant mice and demonstrated that ICAM-1 released from macrophages of calcification-prone mice can increase the calcification of mesenchymal stem cells.

Macrophages from C3H mice were more prone to polarization and expressed higher levels of M1 and M2 markers than those from B6 mice. Expression of *Arg-1*, an M2 marker, was observed only in macrophages derived from splenic monocytes of C3H mice. Similarly, the complement factors *C3a* and *C3ar* were expressed at higher levels in monocytes and macrophages from C3H mice than in those from B6 mice. OCs seem to influence OB differentiation via C3a, which activates the complement system^46^. C3- and C5-deficient mice exhibit reduced bone formation in the early healing phase^47^. Furthermore, ICAM-1, which is chemotactic for a wide variety of immune cells, was released from C3H macrophages *in vitro*, suggesting that macrophage aggregation in the injured heart tissue of C3H mice may occur in response to ICAM-1 release. ICAM-1 is expressed in mature osteoblasts inducing RANKL-secretion, adhesion of osteoclast precursors and thus osteoclast maturation^48^. The role of ICAM-1 in osteoclastogenesis has been reported previously^49–51^. In particular, Suzuki and co-workers demonstrated that neutralization of ICAM-1 inhibited osteoclastogenesis of SW982 cells *in vitro*^51^. Kurashi *et al*. already published results in 1993 indicating that ICAM-1 may play a role in osteoclast development via interaction between stromal cells and osteoclast progenitors as well as among osteoclast progenitors. Okada *et al*. propose that ICAM-1-mediated cell-to-cell adhesion of osteoblasts and osteoclast precursors is involved in RANKL-dependent osteoclast maturation stimulated by 1,25D, PTH, and IL-1-alpha^48^. Additionally, ICAM-1 deficiency protects against atherosclerosis in mice^21^.

Thus, future studies will target ICAM-1 *in vivo*, to determine whether this molecule protects against cardiac calcification.

Our *in vitro* data provide further evidence that exogenous PPi inhibits calcification in C3H mice by influencing the differentiation of MN cells. The formation of MN cells from B6 macrophages did not increase in the presence of serum from C3H mice. These results indicate that the DCC-resistant B6 mice are genetically resistant to MN formation and calcification. However, C3H mice, in which Abcc6 function is altered, are genetically predisposed to MN cells formation, and thus initiate calcification after injury. Thus, we hypothesized that targeting MN cells formation with PPi or other drugs may be an effective therapeutic approach to prevent calcification in C3H mice.

We next showed that administration of PPi inhibited cardiac calcification in response to freeze–thaw injury *in vivo*, in two different calcification-prone mouse models (*Abcc6*^−/−^ B6 mice and C3H mice). Thus, BiPs, analogues of PPi, may have potential as inhibitors of soft tissue calcification. Cardiac calcification is not an uncommon pathology^52–56^. In light of our findings and in parallel with this work, we very recently demonstrated that etidronate prevents cardiac calcification^25^. A partial inhibition of cardiac calcification was observed in mice treated with PPi, but a strong inhibition was seen in mice treated with etidronate. To further test whether calcification is reversible, mice were treated with etidronate after calcification had begun, at day 4 post-injury. In this experiment, no dramatic inhibition of calcification was observed. Interestingly, a single injection of etidronate on day 3, when macrophage infiltration and aggregation is evident in injured tissue, reduced calcification dramatically. This finding demonstrates the key role of macrophage infiltration, aggregation, and fusion in the calcification process. A single dose treatment at defined time of macrophage infiltration may be proposed as therapeutic approach rather than the daily treatment, if one takes in account the bad side effect of BiPs.

**Figure 10:**
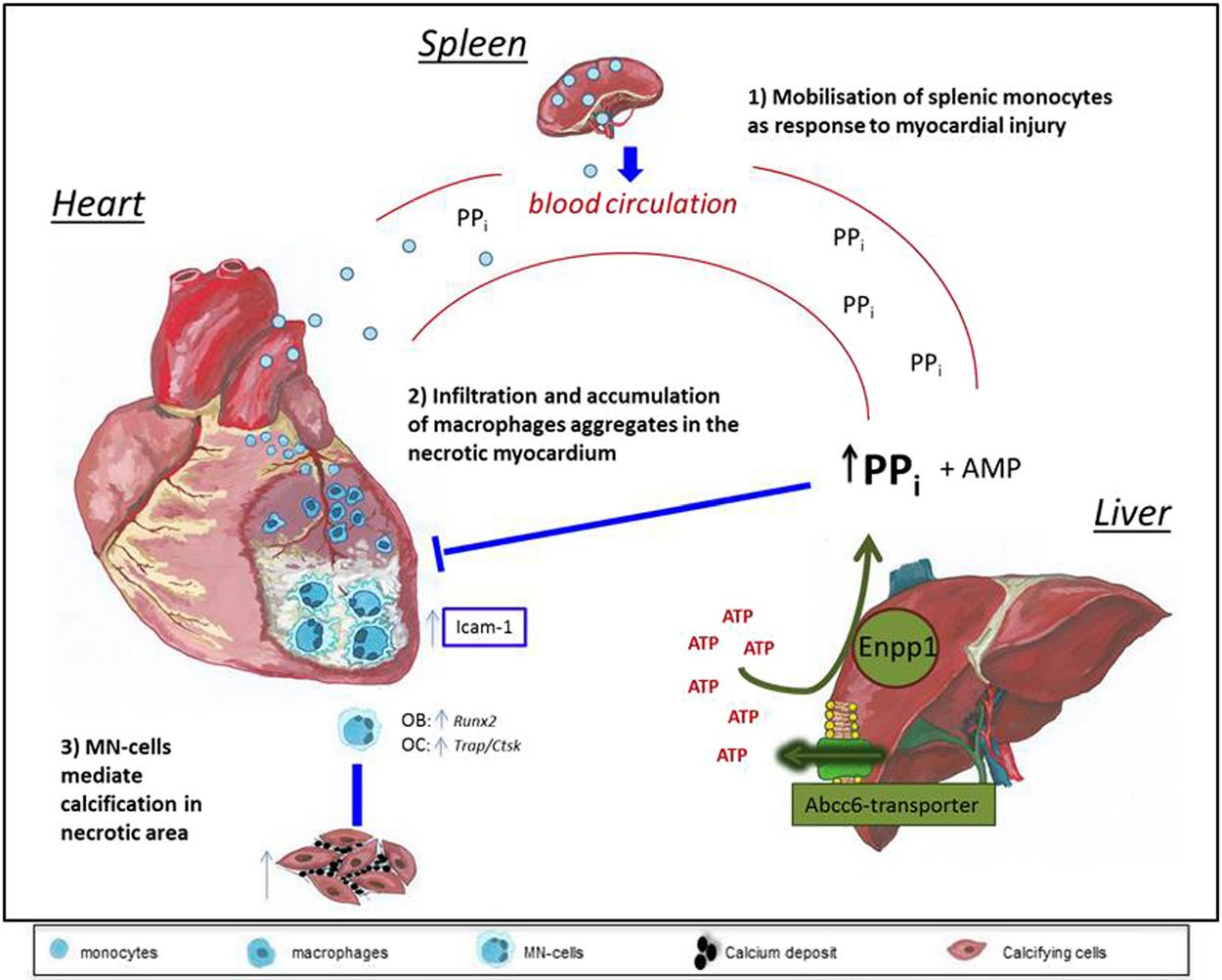
Proposed model: Abcc6 prevents DCC by inhibiting osteoclastogenesis. In our mouse model, tissue damage in the cardiovascular system in response to freeze–thaw injury of the heart induces an inflammatory response involving cross-talk between organs, mainly the liver and heart, mediated by the circulating blood. Hematopoietic stem cells in the bone marrow are mobilized and circulate to the spleen, which serves as a monocyte reservoir and site of monocyte differentiation (1). Circulating monocytes infiltrate and accumulate in necrotic heart tissue and differentiate into macrophages under the influence of local inflammatory mediators such as ICAM-1 (2). Subsequently, macrophages aggregate and fuse to form multinucleated (MN) cells, which are potentially relevant in calcification processes leading to DCC (3). DCC-susceptible mice exhibit elevated formation of MN cells expressing osteoclastic (OC) and osteoblastic (OB) markers. MN cells generate calcium deposits involving cardiac cells. *Abcc6*, which is mainly expressed in hepatocytes, encodes a membrane transporter and is the causal gene for DCC in mice. The functional Abcc6 transporter promotes the release of ATP, which is converted into AMP and inorganic pyrophosphate (PPi) via Enpp1 (ectonucleotide pyrophosphatase/phosphodiesterase). PPi is a potent inhibitor of osteoclastogenesis and smooth muscle cell calcification. High circulating levels of PPi in DCC-resistant mice or supplementation of DCC-susceptible mice with PPi inhibits macrophage differentiation into MN cells, and thus prevents formation of calcium deposits (3).

In summary, these data demonstrate that mice predisposed to calcification are deficient in circulating PPi and exhibit higher macrophage secretion of ICAM-1, leading to further macrophage aggregation and calcium phosphate deposition (Fig. 10). Exogenous administration of PPi or agents targeting ICAM-1 may be possible therapeutic approaches to prevent calcification.

## Electronic supplementary material

Supplementary Information
