# Supplementary material for: Etidronate prevents dystrophic cardiac calcification by inhibiting macrophage aggregation
Source: Sci Rep. 2018 Apr 11;8:5812. doi: 10.1038/s41598-018-24228-y (PMC5895639; doi:10.1038/s41598-018-24228-y)
Supplement: Supplementary file 1 — Supplementary Information [file 41598_2018_24228_MOESM1_ESM.docx]

**Etidronate prevents dystrophic cardiac calcification by inhibiting macrophage aggregation**

Carolin Bauer (1), Olivier le Saux (2), Viola Pomozi (2), Redouane Aherrahrou (1, 3), Rene Kriesen (1), Stephanie Stölting (1), Annett Liebers (1), Thorsten Kessler (4), Heribert Schunkert (4), Jeanette Erdmann (1) *, Zouhair Aherrahrou (1) *

**SUPPLENTARY UNPROCESSED ORIGINAL SCANS for figure 4, C.**

**Figure 4,** C: Images of C5a expression in monocytes, macrophages, MN and liver tissue: C5a expression was not detected in monocytes (Mo), macrophages (Ma), or MN cells of B6 or C3H mice. Liver tissue was used as a positive control where C5a is highly expressed. From left to right: Marker (100bp), B6-Liver, B6-Mo, B6-Ma, B6-MN, C3H-Liver, C3H-Mo, C3H-Ma, C3H-MN, H2O, Marker

**Figure 4,** C: Images of Beta-Actin expression in monocytes, macrophages, MN and liver tissue. Beta-Actin expression was detected in all samples. From left to right: Marker (100bp), B6-Liver, B6-Mo, B6-Ma, B6-MN, C3H-Liver, C3H-Mo, C3H-Ma, C3H-MN and H2O.
